# Antifungal Effects of Pterostilbene on *Candida albicans*, *Candida dubliniensis*, and Microcosm Biofilms of Denture Stomatitis

**DOI:** 10.3390/jof11120869

**Published:** 2025-12-07

**Authors:** Paulo Henrique Fonseca do Carmo, Mirian de Fátima da Silva, Amanda Siqueira Fraga, Juliana Caparroz Gonçale, Patrícia Michelle Nagai de Lima, Geovani Moreira da Cruz, Karoline Kristina Kemmerich, Felipe de Camargo Ribeiro, Maíra Terra Garcia, Juliana Campos Junqueira

**Affiliations:** 1 Department of Biosciences and Oral Diagnosis, Institute of Science and Technology, São Paulo State University (Unesp), Av. Engenheiro Francisco José Longo, 777, Jardim São Dimas, São José dos Campos 12245-000, SP, Brazil; phf.carmo@unesp.br (P.H.F.d.C.);; 2 Departament of Genetics, Microbiology and Immunology, Botucatu Institute of Biosciences, São Paulo State University (Unesp), Botucatu 18618-689, SP, Brazil; 3 Special Mycology Laboratory, Paulista School of Medicine, Federal University of São Paulo (Unifesp), São Paulo 04023-062, SP, Brazil

**Keywords:** polyphenol, *Candida* spp., biofilms, denture stomatitis, *Galleria mellonella*

## Abstract

Natural compound-based strategies have gained attention as alternatives to conventional antifungal therapies, particularly in the management of *Candida* infections affecting the oral cavity, such as denture stomatitis. Our aim was to investigate the antifungal activity of the polyphenol pterostilbene (PTE) on clinical *Candida* isolates and microcosm biofilms from denture stomatitis, as well as to evaluate its toxicity and therapeutic efficacy in *Galleria mellonella*. PTE exhibited fungicidal effects against *Candida albicans* and *Candida dubliniensis* at 32 µg/mL. Time-kill assays demonstrated complete inhibition of viability for both strains within 8 h of exposure. In addition, PTE exhibited broad-spectrum antimicrobial activity, significantly reducing the counts of streptococci, *mutans* streptococci, staphylococci, and yeasts within microcosm biofilms. In vivo, PTE showed no signs of toxicity in *G. mellonella* at concentrations up to 20× MIC. Prophylactic treatment with PTE enhanced larval survival in experimental candidiasis caused by both *C. albicans* and *C. dubliniensis*. Moreover, prophylactic treatment decreased the fungal burden of *C. albicans* in the *G. mellonella* hemolymph, while the recruitment of hemocytes involved in host defense mechanisms remained unaltered. In summary, PTE demonstrated antimicrobial activity against *Candida* planktonic cells and complex biofilms associated with denture stomatitis, exhibiting favorable biocompatibility and in vivo antifungal efficacy in *G. mellonella* model.

## 1. Introduction

The *Candida* genus comprises yeast species that are part of the normal human microbiota and are commonly found on the skin, in the gastrointestinal and genitourinary tracts, and in the oral cavity [1]. Under healthy conditions, *Candida* spp. behaves as commensal. However, disruptions in host homeostasis—such as compromised immune defenses or factors like broad-spectrum antibiotic use, *diabetes mellitus*, chemotherapy, or the use of medical and dental devices (e.g., catheters and prostheses)—can promote yeast overgrowth [2,3]. These conditions may shift *Candida* from a commensal to a pathogenic state, leading to infections of the skin and mucous membranes, and potentially to hematogenous dissemination [4,5].

*Candida* colonization and infection are driven by several fungal virulence factors, including morphological transitions, adhesion and filamentation capabilities, secretion of hydrolytic enzymes, and biofilm formation [6,7]. Among these, biofilm formation plays a critical role. Biofilms are complex, structured communities of microorganisms embedded in a self-produced extracellular matrix. In the oral cavity, *Candida* infections are closely linked to the yeast’s ability to adhere and form biofilms on both biotic (oral mucosa) and abiotic (dentures) surfaces [8,9]. This process is a major contributor to *Candida*-associated denture stomatitis—a condition affecting from 20 to 75% of denture wearers [10,11].

Conventional treatment of *Candida* spp. infections primarily rely on antifungal agents from the polyene, azole, and echinocandin classes [12]. However, prolonged use of these drugs presents several limitations, including hepatotoxicity and nephrotoxicity, limited bioavailability, and the emergence of resistant strains [13,14,15]. For example, resistance to fluconazole, a widely used antifungal for managing *Candida* infections, has been estimated to range between 0.5% and 13% among *Candida* species [16]. To overcome these challenges, the search for alternative therapies—particularly those based on natural compounds such as polyphenols—has gained increasing attention [17].

Polyphenols are low molecular weight organic compounds characterized by one or more phenolic groups and are widely distributed in various plant species. In humans, they exhibit a range of biological activities, including anticancer, anti-inflammatory, antioxidant, and antimicrobial effects. These therapeutic properties are largely attributed to their antioxidant properties, which include the ability to reduce reactive oxygen and nitrogen species, enhance the enzymatic activity of the antioxidant system, and mitigate inflammation [18,19].

Among these, pterostilbene has emerged as a promising polyphenol with notable antimicrobial properties. It belongs to the stilbene class and is characterized by high lipophilicity and a broad spectrum of pharmacological activities. Naturally occurring in fruits such as blueberries, grapes, and plums, pterostilbene is synthesized by plants as a secondary metabolite in response to environmental stressors, including pathogen invasion and excessive ultraviolet radiation exposure [20]. The antibacterial activity of pterostilbene has been demonstrated against *Pseudomonas aeruginosa*, *Escherichia coli*, and *Staphylococcus aureus* [21,22]. In addition, pterostilbene has shown antifungal effects against filamentous fungi such as *Aspergillus flavus* [23] and yeasts including *Candida albicans* [24]. However, the effects of PTE on complex heterotypic biofilms and its therapeutic efficacy in vivo remain unexplored.

In this study, we investigated the antifungal activity of pterostilbene against clinical isolates of *Candida* spp. and microcosm biofilms associated with denture stomatitis. Furthermore, we evaluated its antifungal and immunomodulatory effects in an in vivo experimental candidiasis model using the invertebrate host *Galleria mellonella*.

## 2. Materials and Methods

### 2.1. Clinical Samples

In this study, clinical samples were collected from the lesions of denture stomatitis of two patients (P1 and P2). From these samples, *Candida* strains were isolated and identified by mass spectrometry (MALDI-TOF). The isolates of patient 1 and patient 2 were identified as *Candida dubliniensis* (strain P1) and *Candida albicans* (strain P2), respectively. In addition, both clinical samples of patient 1 (sample 1) and patient 2 (sample 2) were used to form microcosm biofilms.

The collections were approved by the Human Research Ethics Committee of the Institute of Science and Technology of São Paulo State University (ICT/UNESP) under number 5.827755. These strains and samples are kept frozen in a freezer at −80 °C in the Oral Microbiology and Immunology Laboratory (ICT/UNESP).

### 2.2. Activation of Candida Strains and Pterostilbene Preparation

*Candida dubliniensis* P1 and *C. albicans* P2 were cultured on Sabouraud dextrose agar (SDA; Kasvi, Pinhais, PR, Brazil) at 37 °C for 24 h prior to each assay. The polyphenol pterostilbene (PTE; Sigma-Aldrich, St. Louis, MI, USA) and the antifungal fluconazole (FCZ; Sigma-Aldrich) were used. Stock solutions of PTE and FCZ at 10,000 µg/mL were prepared in dimethyl sulfoxide (DMSO; Sigma-Aldrich).

### 2.3. Susceptibility Assay

The susceptibility of *Candida* spp. to PTE and FCZ was determined using the broth microdilution method according to manual E.Def 7.3.2 of the European Committee on Antimicrobial Susceptibility Testing (EUCAST). Stock solutions of PTE and FCZ were diluted in RPMI 1640 (Sigma-Aldrich) culture medium with L-glutamine, pH 7.0, without sodium bicarbonate, supplemented with 2% glucose, and buffered with 0.165 M 3-(N-morpholino) propanesulfonic acid (MOPS; Sigma-Aldrich). We used concentrations ranging from 0.5 to 512 µg/mL for PTE, and from 0.12 to 64 µg/mL for FCZ.

Fungal inoculum was prepared from cultures of *C. dubliniensis* P1 and *C. albicans* P2 on SDA for 24 h at 37 °C. The cells were suspended in distilled water, homogenized, and standardized to a final concentration of 5.0 × 10^5^ cells/mL. Microplates containing 100 µL of 2-fold serial dilutions of PTE and FCZ were inoculated with 100 µL of the adjusted inoculum. Growth (100 µL of RPMI + 100 µL of fungal inoculum) and sterility (100 µL of RPMI + 100 µL of distilled water) controls were included. The reference strain *Pichia kudriavzevii* (formerly *Candida krusei*) ATCC 6258 was used as quality control. After incubation at 37 °C for 24 h, the absorbance of microplates was measured in a spectrophotometer (Epoch, Biotek Instruments, Winooski, VT, USA) at 530 nm. The minimum inhibitory concentration (MIC) was considered as the lowest concentration capable of inhibiting at least 90% of fungal growth for PTE, and 50% for FCZ, compared to the growth control.

For the minimum fungicidal concentration (MFC) assay, 10 μL was removed from each well without visible growth, cultured on SDA plates, and incubated for 24 h at 37 °C. The minimum fungicidal concentration (MFC) was determined as the lowest concentration of PTE capable of preventing 100% fungal growth after plating. Then, the MFC/MIC ratio was performed. PTE was considered fungicidal if the MFC/MIC ratio was ≤4 and fungistatic if the ratio was >4 [25].

### 2.4. Time-Kill Curves

Time-kill assays were performed using the RPMI medium supplemented with 2% glucose as the growth medium. Fungal inoculum was prepared from *C. dubliniensis* P1 and *C. albicans* P2 cultures diluted in distilled water, quantified, and adjusted to a final concentration of 2.5 × 10^5^ cells/mL. Then, both strains were treated with PTE at MIC, followed by incubation at 37 °C. Non-treated cells were used as negative controls. Aliquots were taken at 0, 2, 8, 24, and 48 h after exposure and plated on SDA to determine the colony-forming units (CFU/mL).

### 2.5. Effects of PTE on Microcosm Biofilms of Denture Stomatitis

Microcosm biofilms were formed from clinical samples P1 and P2, collected from two volunteers clinically diagnosed with oral candidiasis associated with denture use. Cervical brushes were used to collect specimens from both the palatal lesion and the surface of the dentures, which were then stored in brain heart infusion (BHI, Kasvi, Paraná, Brazil) broth. The presence of yeast cells suggestive of *Candida* spp. was confirmed through microscopic analysis and by growth on SDA supplemented with 0.5% chloramphenicol, as well as on a chromogenic selective medium (Chromagar *Candida* Plus; Difco, Franklin Lakes, NJ, USA) for presumptive identification of *Candida* species.

After collection, both samples were thawed and homogenized. Subsequently, 400 μL of each sample was added to 20 mL of fresh BHI broth with 5% sucrose and incubated at 37 °C with 5% CO_2_ for 24 h. After this period, the suspension was centrifuged (5000 rpm) for 10 min, the supernatant was discarded, and the cells were resuspended in 10 mL of fresh BHI broth with 5% sucrose. Then, 225 µL of the previously grown sample was added to each well, and the microplates were incubated at 37 °C for 96 h with 5% CO_2_. The wells were washed, and 225 µL of fresh BHI broth with 5% sucrose was added every 24 h.

After biofilm formation, the microcosm biofilms were treated for 24 h with PTE at 1×, 5×, 10× and 20× MIC. Non-treated groups were used as negative controls. Then, the biofilms were disrupted using an ultrasonic homogenizer (Sonoplus HD2200, Bandelin Electronic, Berlin, Germany) with a power of 7 W for 30 s. From the solution obtained, serial dilutions were performed and plated in five different culture media: BHI for non-selective counting, Mitis Salivarius agar (MS; Difco) for counting streptococci, Mitis Salivarius Bacitracin Sucrose agar (MSBS; Difco) for counting *mutans* streptococci, Mannitol agar (MAN; Difco) for counting staphylococci, and SDA with chloramphenicol for counting yeasts. The plates were then incubated at 37 °C for 24 h under 5% CO_2_ to determine microbial viability (CFU/mL) [26].

### 2.6. Standardization of Candida Inoculation Conditions and PTE Treatment in G. mellonella

To establish a model of experimental infection with *C. dubliniensis* P1 and *C. albicans* P2, as well as the PTE treatment protocol in *G. mellonella*, we evaluated the toxicity of PTE and the effects of infection at varying *Candida* concentrations.

To assess toxicity, PTE was injected at different concentrations (1×, 10× and 20× MIC) into the last proleg of *G. mellonella* larvae (n = 10) using a 10 µL 26G Hamilton syringe (Hamilton Company, Reno, NV, USA). A group inoculated with PBS was included as a control. Larvae were monitored for 5 days to generate survival curves. Larvae were considered dead if they did not respond to touch [27].

To determine the appropriate inoculum for *Candida* strains, *G. mellonella* larvae (n = 10) were inoculated with three different concentrations of each strain (5 × 10^7^, 1 × 10^8^ and 5 × 10^8^) using a Hamilton syringe. A group inoculated with PBS was included as control. Larvae were then monitored for 5 days, and those that did not respond to touch were considered dead [27].

### 2.7. Study of the Prophylactic and Therapeutic Effects of PTE in Experimental Candidiasis in G. mellonella: Analysis of Survival Curves

Since the toxicity of PTE on *G. mellonella* larvae and the inoculum concentration were established, two different treatment approaches were evaluated. In the first approach (prophylactic), larvae were pre-treated with PTE at 20× MIC prior (24 h before) to infection with *C. dubliniensis* P1 or *C. albicans* P2 (1 × 10^8^ cells/mL). In the second approach (therapeutic), larvae were treated with PTE at 20× MIC after (1 h) infection with *C. dubliniensis* P1 or *C. albicans* P2 (1 × 10^8^ cells/mL). Treatment with PTE was inoculated in the last right proleg, while *Candida* spp. was injected in the last left proleg using a 10 µL 26G Hamilton syringe. Then, larvae were incubated at 37 °C and monitored for 5 days. Larvae were considered dead if they did not respond to touch [27].

### 2.8. Study of Prophylactic Effects of PTE in Experimental Candidiasis in G. mellonella: Analysis of Fungal Burden and Hemocyte Recruitment

Since the prophylactic approach exhibited promising results compared to the therapeutic, we assessed the effects of PTE treatment in fungal burden and hemocyte recruitment. Thus, larvae (n = 12) were inoculated with PTE at 20× MIC prior (24 h before) inoculation with *C. dubliniensis* P1 or *C. albicans* P2, both at 1 × 10^8^ cells/mL. Control groups with larvae inoculated with *C. dubliniensis* P1 or *C. albicans* P2 and not treated were included. After 1 h of infection with *Candida* spp., larvae were cut ventrally in the cephalocaudal direction, and the hemolymph was diluted in saline solution for insects (IPS; 2% NaCl; glucose 0.1 M; sodium citrate 30 mM; citric acid 26 mM and EDTA 10 mM).

To study the antifungal effects of PTE, the hemolymph of the larvae (n = 6) was diluted and plated in ASD. After incubation for 24 h at 37 °C, the fungal burden in the hemolymph (CFU/mL) was determined [26]. To investigate the immunomodulatory effects of PTE, 10 μL of the hemolymph from each larva (n = 6) was dispensed into a hemocytometer and the number of hemocytes for each experimental group was quantified [27].

### 2.9. Statistical Analysis

Statistical analysis was performed using GraphPad Prism, version 6.0, for Windows (GraphPad Software, San Diego, CA, USA), with *p* < 0.05 considered significant. The results of CFU/mL counting (time-kill curves, microcosm biofilm assays and fungal burden in *G. mellonella*) and hemocyte counting were analyzed by ANOVA, followed by Tukey’s test to compare different groups. The survival curve of *G. mellonella* larvae was analyzed using the Kaplan–Meier method and the Log-rank test (Mantel–Cox).

## 3. Results

### 3.1. PTE Exhibited Fungicidal Effects on Candida Planktonic Cells

In the broth microdilution tests, the MIC of PTE was 32 μg/mL for both *Candida* strains. The MIC of FCZ was 0.25 μg/mL for *C. dubliniensis* P1 and *C. albicans* P2, and 16 μg/mL for the quality control strain (*P. kudriavzevii* ATCC 6258). The MFC was 32 μg/mL for *C. dubliniensis* P1 and *C. albicans* P2, with an MFC/MIC ratio of 1, confirming the fungicidal activity of PTE on both *Candida* strains (Table 1).

**Figure 1:**
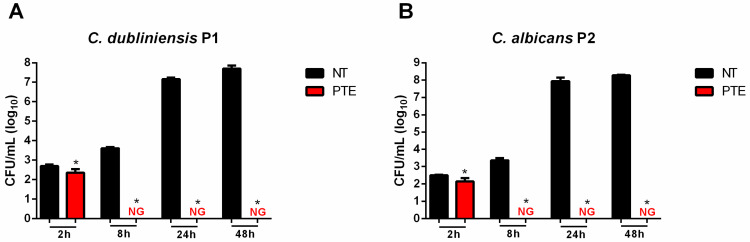
**Treatment with pterostilbene (PTE) inhibited *Candida* growth.** Viability (log_10_ CFU/mL) of (**A**) *Candida dubliniensis* P1 and (**B**) *C. albicans* P2 non-treated (NT) and treated with PTE for 48 h. NG: no growth. * Significant difference (*p* < 0.05) compared to the NT group.

Time-kill curve analysis demonstrated the inhibitory effects of PTE against *Candida* strains. PTE reduced the growth of *C. dubliniensis* P1 and *C. albicans* P2 within 2 h (Figure 1). Furthermore, complete inhibition of both strains was observed after 8 h of treatment with PTE. Importantly, the inhibitory effects persisted throughout the 48 h analysis, confirming its fungicidal effects on both *Candida* strains (Figure 1).

### 3.2. PTE Demonstrated Broad Antimicrobial Activity Against Microcosm Biofilms Associated with Denture Stomatitis

**Figure 2:**
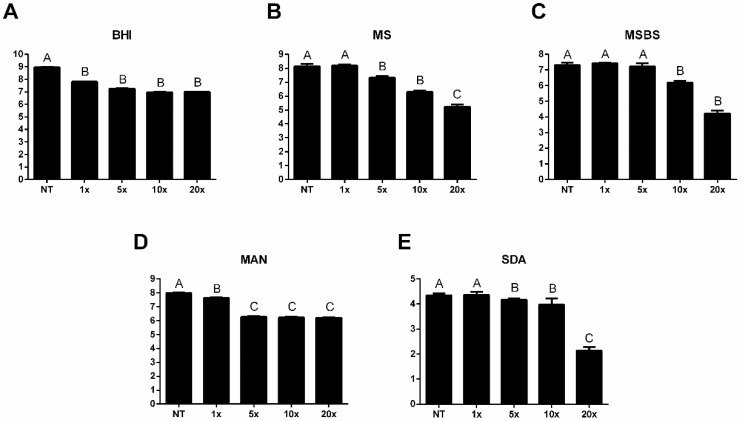
**Pterostilbene (PTE) reduced the microbial viability of the microcosm biofilm formed from clinical sample P1.** Microbial viability (log_10_ CFU/mL) of microcosm biofilms from the sample P1, either non-treated (NT) and treated with pterostilbene (PTE) at 1×, 5×, 10×, and 20× the minimum inhibitory concentration (MIC), plated on (**A**) Brain Heart Infusion (BHI), (**B**) Mitis Salivarius (MS), (**C**) Mitis Salivarius Bacitracin Sucrose (MSBS), (**D**) Mannitol (MAN), and (**E**) Sabouraud dextrose (SDA) media. Different letters represent a statistically significant difference (*p* < 0.05).

Since PTE exhibited effects on *Candida* planktonic cells, we investigated its activity on denture stomatitis microcosm biofilms formed from patient 1 (sample P1) and patient 2 (sample P2). Treatment of biofilms from sample P1 with PTE reduced the microbial count in BHI, MS, MSBS, MAN, and ASD. In the non-selective microorganism count (BHI), a reduction of 1.15 log_10_ CFU/mL was observed following treatment with PTE at 1× MIC. At concentrations of 5×, 10×, and 20× MIC, the reductions were 1.71, 2.00, and 1.98 log_10_ CFU/mL, respectively (Figure 2A). Regarding the streptococci count (MS), microbial viability was decreased in a dose-dependent manner, with reductions observed at 5× (0.77 log_10_ CFU/mL), 10× (1.80 log_10_ CFU/mL), and 20× MIC (2.88 log_10_ CFU/mL) (Figure 2B). For the *mutans* streptococci group (MSBS), treatment with PTE at 10× MIC resulted in a reduction of 1.04 log_10_ CFU/mL, while at 20× MIC, the decrease was 3.04 log_10_ CFU/mL (Figure 2C). Staphylococcal growth (MAN) was reduced at all tested concentrations of PTE, ranging from 0.37 log_10_ CFU/mL at 1× MIC to 1.80 log_10_ CFU/mL at 20× MIC (Figure 2D). The yeast count (SDA) was significantly reduced after treatment with PTE at 5×, 10× and 20× MIC, with reductions of 0.16, 0.49, and 2.21 log_10_ CFU/mL, respectively (Figure 2E).

**Figure 3:**
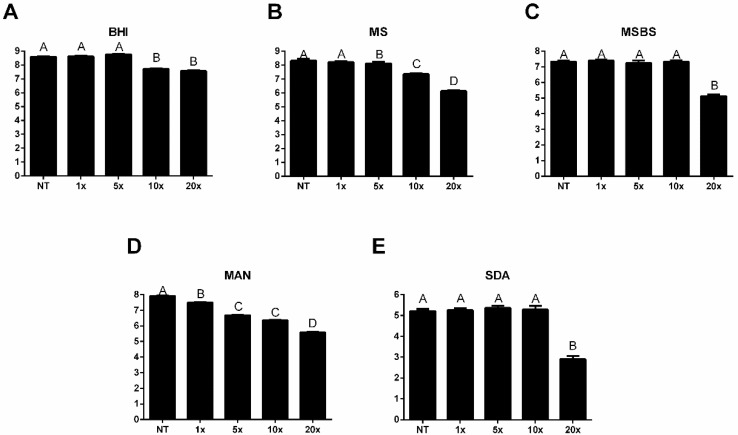
**Pterostilbene (PTE) reduced the microbial viability of the microcosm biofilm formed from clinical sample P2.** Microbial viability (log_10_ CFU/mL) of microcosm biofilms from the sample P2, either non-treated (NT) and treated with pterostilbene (PTE) at 1×, 5×, 10×, and 20× the minimum inhibitory concentration (MIC), plated on (**A**) Brain Heart Infusion (BHI), (**B**) Mitis Salivarius (MS), (**C**) Mitis Salivarius Bacitracin Sucrose (MSBS), (**D**) Mannitol (MAN), and (**E**) Sabouraud dextrose (SDA) media. Different letters represent a statistically significant difference (*p* < 0.05).

PTE also decreased microbial viability of the microcosm biofilm formed from clinical sample P2 across all tested media, with significant reductions observed at 10× and 20× MIC. Treatment with PTE significantly decreased the microbial count in non-selective media (BHI), with reductions of 0.85 and 1.02 log_10_ CFU/mL at 10× and 20× MIC, respectively (Figure 3A). In the streptococci count (MS), reductions of 0.19 log_10_ CFU/mL at 5× MIC, 0.97 log_10_ CFU/mL at 10× MIC, and 2.18 log_10_ CFU/mL at 20× MIC were observed (Figure 3B). While the mean CFU count for *mutans* group streptococci (MSBS) in the non-treated (NT) group was 7.33 log_10_ CFU/mL, the count in the group treated with PTE at 20× MIC decreased to 5.12 log_10_ CFU/mL (Figure 3C). For staphylococci counts (MAN), viability was reduced at PTE concentrations of 1×, 5×, 10×, and 20× MIC, with mean reductions of 0.42, 1.23, 1.57, and 2.33 log_10_ CFU/mL, respectively. (Figure 3D). The yeast count (SDA) was significantly reduced following treatment with PTE at 20× MIC, resulting in a decrease of 2.30 log_10_ CFU/mL (Figure 3E).

### 3.3. G. mellonella Larvae Did Not Have Toxic Reactions After PTE Administration, and Were Susceptible to Infections Caused by Clinical Candida Isolates

Toxicity analysis revealed that *G. mellonella* larvae maintained full viability following inoculation with PTE at concentrations up to 20× MIC. Based on these findings, we selected the 20× MIC for subsequent assays using *G. mellonella* larvae.

**Figure 4:**
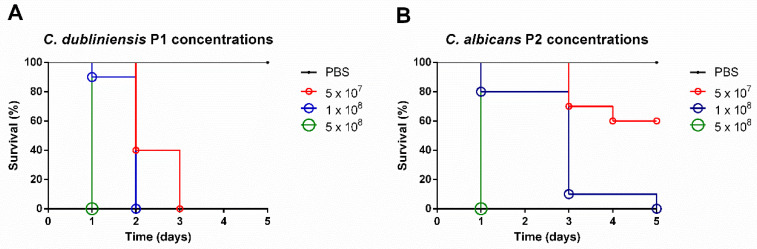
***Candida* strains caused experimental infection in *G. mellonella* larvae.** Survival curves of *G. mellonella* larvae inoculated with PBS, (**A**) *C. dubliniensis* P1 and (**B**) *C. albicans* P2 at 5 × 10^7^, 1 × 10^8^ and 5 × 10^8^ cells/mL.

In the experimental infection of *G. mellonella* with *C. dubliniensis* P1, 100% of larvae inoculated with 5 × 10^8^ cells/mL succumbed within one day. In the group inoculated with 1 × 10^8^ cells/mL, 10% of larvae died on day 1, while mortality reached 100% by day 2. In addition, 60% of larvae inoculated with 5 × 10^7^ cells/mL died by day 2, and the remaining 40% succumbed on day 3 (Figure 4A).

For *C. albicans* P2, 100% mortality occurred within one day for larvae inoculated with 5 × 10^8^ cells/mL. In the group inoculated with *C. albicans* P2 at 1 × 10^8^ cells/mL, 100% mortality was observed by day 5, while 60% of larvae inoculated at 5 × 10^7^ cells/mL remained viable throughout the five-day monitoring period (Figure 4B). Based on these results, the standardized inoculum for experimental infection in *G. mellonella* larvae was established at 1 × 10^8^ cells/mL for both *Candida* strains.

### 3.4. Prophylactic Treatment with PTE Increased the Survival Rate of G. mellonella Larvae Infected by Candida Strains

**Figure 5:**
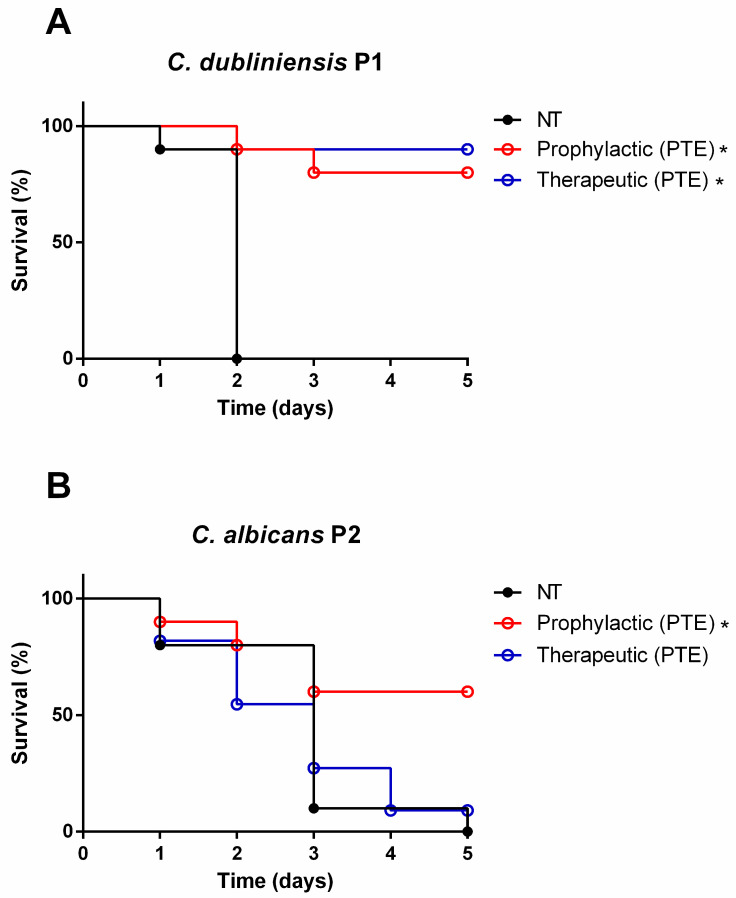
**Prophylactic treatment with pterostilbene (PTE) improved the survival of *G. mellonella* larvae following experimental infection with *Candida* spp.** (**A**) Survival curves of *G. mellonella* larvae pre-treated PTE (prophylactic PTE) at 20× minimum inhibitory concentration (MIC), followed by inoculation with *Candida dubliniensis* P1 (1 × 10^8^ cells/mL). Additional groups included larvae inoculated with *C. dubliniensis* P1 and either non-treated (NT), and post-treated with PTE (therapeutic PTE). (**B**) Survival curves of *G. mellonella* larvae pre-treated with PTE (prophylactic PTE) at 20× MIC, followed by inoculation with *Candida albicans* P2 (1 × 10^8^ cells/mL). Additional groups included larvae inoculated with *C. albicans* P2 and either non-treated (NT), and post-treated with PTE (therapeutic PTE). * Significant difference (*p* < 0.05) compared to the NT group.

To investigate the in vivo antifungal and immunomodulatory effects of PTE, we evaluated both prophylactic and therapeutic strategies in an experimental model of candidiasis using *G. mellonella*. In infections with *C. dubliniensis* P1, no mortality was observed in the prophylactic group on day 1, while the therapeutic group showed 10% mortality. By day 5, mortality rates reached 20% in the prophylactic group and 10% in the therapeutic group. Both treatment strategies significantly improved larvae survival (Figure 5A).

In infections with *C. albicans* P2, prophylactic treatment resulted in 10% larval mortality on day 1, whereas therapeutic treatment led to 20% mortality. By day 5, mortality increased to 40% in the prophylactic group and reached 90% in the therapeutic group. Prophylactic administration of PTE significantly enhanced larval survival, while the therapeutic approach showed no significant protective effect (Figure 5B).

### 3.5. Prophylactic Treatment with PTE Reduced Fungal Burden in G. mellonella Infected with C. albicans Without Alter the Hemocyte Count

**Figure 6:**
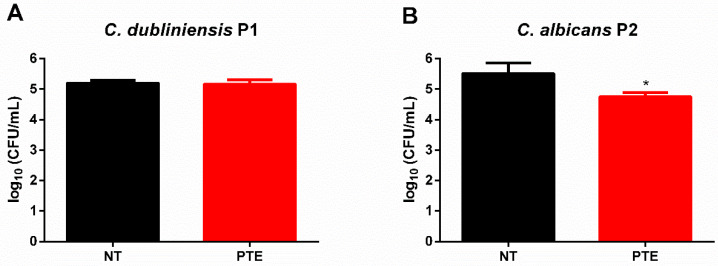
**Prophylactic treatment with pterostilbene (PTE) reduced fungal burden in *G. mellonella* infected with *Candida albicans.*** (**A**) Fungal burden (CFU/mL) in the hemolymph of larvae infected with *C. dubliniensis* P1 and either non-treated (NT) or pre-treated with PTE (PTE). (**B**) Fungal burden (log_10_ CFU/mL) in the hemolymph of larvae infected with *C. albicans* P2 and either non-treated (NT) or pre-treated with PTE (PTE). * Significant difference (*p* < 0.05) compared to the NT group.

Given the superior outcomes observed with the prophylactic approach compared to the therapeutic strategy, we assessed its effects on fungal burden in the hemolymph of *G. mellonella* larvae and on hemocyte recruitment. Prophylactic treatment with PTE had no effect on fungal burden in the hemolymph of *G. mellonella* larvae infected with *C. dubliniensis* P1 (Figure 6A). However, in larvae pre-treated with PTE and infected with *C. albicans* P2, a significant reduction in fungal burden was observed (Figure 6B).

**Figure 7:**
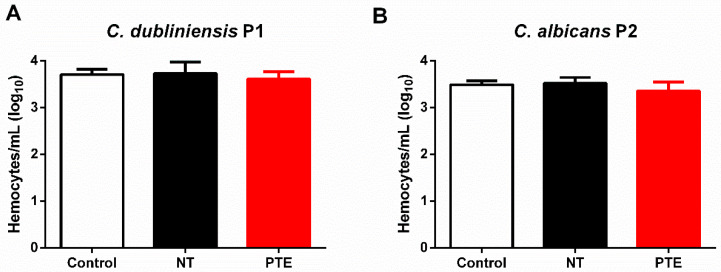
**Prophylactic treatment with pterostilbene (PTE) did not impact the hemocyte counting in *G. mellonella* larvae.** (**A**) Hemocyte counting (hemocytes/mL) in the hemolymph of larvae inoculated with PBS (control), infected with *C. dubliniensis* P1 and either non-treated (NT) or pre-treated with PTE (PTE). (**B**) Hemocyte counting (hemocytes/mL) in the hemolymph of larvae inoculated with PBS (control), infected with *C. albicans* P2 and either non-treated (NT) or pre-treated with PTE (PTE).

Subsequently, the immunomodulatory effects of PTE on *G. mellonella* were analyzed. Prophylactic treatment with PTE did not affect hemocyte counting compared to the control and non-treated groups for both *Candida* strains (Figure 7).

## 4. Discussion

The treatment of oral *Candida* infections has been hindered by limitations in the current antifungal arsenal, stimulating the search for new antifungal agents [13,15,28]. In this study, we investigated the antifungal effects of PTE against *C. albicans* and *C. dubliniensis* associated with denture stomatitis using both in vitro and in vivo approaches.

The effects of PTE were initially investigated on planktonic cells of *Candida*. Microdilution assays confirmed its fungicidal effects against *C. dubliniensis* and *C. albicans* at 32 μg/mL. Previous studies have reported similar inhibitory effects of PTE at concentrations ranging from 16 to 32 μg/mL against *C. albicans* and non-*albicans Candida* species, including *Candida tropicalis* and *Nakaseomyces glabratus* (formerly *Candida glabrata*) [29,30]. In addition, Simonetti et al. [29] observed that the treatment with PTE at 32 μg/mL significantly decreased fungal growth after 4 h. In the present study, PTE completely inhibited fungal growth at 8 h of treatment, confirming its fungicidal mode of action.

PTE is a dimethyl ether derivative of the polyphenol resveratrol, distinguished by the methoxyl groups. These structural features confer increased lipophilicity to PTE and enhanced cellular absorption [31]. Due to this chemical similarity, comparisons between the biological activities of PTE and resveratrol have garnered significant scientific interest. However, a previous study by our research group [32] demonstrated that resveratrol failed to inhibit *C. albicans* strains at concentrations up to 256 μg/mL. Moreover, a high fungal burden (10^6^ cells/mL) was still observed at 48 h of resveratrol treatment. Although resveratrol is known for its broad biological activity, our findings indicate that PTE is significantly more effective on planktonic cells, inhibiting *C. albicans* growth at concentrations at least 8× lower. However, further studies are needed to elucidate the mode of action of PTE on fungal cells and to strengthen its potential as a therapeutic agent to overcome drug resistance. Hu et al. [23] reported that PTE treatment led to decreased conidial germination and, consequently, reduced growth of *A. flavus*. Nonetheless, the mode of action of PTE on *Candida* and other fungal species has not yet been investigated.

We subsequently studied the antimicrobial activity of PTE in microcosm biofilms associated with denture stomatitis. Denture stomatitis is a chronic, multifactorial inflammatory condition frequently observed in denture wearers [33]. Clinically, this condition presents as petechiae, diffuse erythema, or granular hyperplasia, typically restricted to the mucosal areas in contact with the prosthesis. These lesions can cause discomfort and burning sensations, significantly impairing the quality of life of affected individuals [11,34]. Denture stomatitis is commonly associated with infections caused by *Candida* spp. and various bacterial pathogens. These microorganisms possess the capacity to form biofilms—complex structures that pose a significant challenge to antifungal therapy due to their heightened resistance [35,36,37]. In our study, PTE exhibited broad-spectrum antimicrobial activity, demonstrated by its ability to reduce the viability of streptococci, staphylococci, *mutans* streptococci, and yeasts. Beyond its relevance to denture stomatitis, these microorganisms are also implicated in other oral infections such as periodontitis and dental caries [38,39].

Previous studies have established the antimicrobial activity of PTE against biofilms of *C. albicans* [24] and staphylococci [21]. PTE was shown to reduce the viability and biomass of *C. albicans* monotypic biofilms at concentrations up to 20 µg/mL [24]. These antibiofilm effects were associated with decreased cellular surface hydrophobicity and inhibition of hyphal formation. Moreover, gene expression analysis confirmed downregulation of key pathways related to morphological transition, ergosterol biosynthesis, antioxidant defense, cell surface structure, and protein folding [30,40]. In a separate study, Vanková et al. [21] reported antibacterial effects of PTE against *Staphylococcus aureus* and *Staphylococcus epidermidis* monotypic biofilms, with minimum biofilm eradication concentrations ranging from 50 to 75 µg/mL. Wang et al. [41] demonstrated that PTE impaired *S. aureus* pathogenesis by targeting critical virulence factors, including β-lactamase biofilms and α-hemolysin. In addition, treatment with PTE elevated reactive oxygen species (ROS) levels against *S. aureus*, leading to cellular damage characterized by irregular morphology, membrane disruption, leakage of nucleic acids and proteins, reduced DNA content, and activation of apoptosis pathways [42].

In the present study, PTE reduced the microbial viability of *C. albicans* and staphylococci at concentrations of 640 and 32 µg/mL, respectively. The discrepancy observed in biofilm inhibitory activity values is likely attributable to the nature of the biofilms examined. While most studies have assessed the effects of PTE on monotypic biofilms, our investigation evaluated its activity against complex heterotypic microcosm biofilms. Microcosm biofilms are in vitro models that simulate the conditions of biofilm formation and development in a controlled setting [43,44,45]. Unlike monotypic models, microcosm systems incorporate biological material from the target environment, capturing the complexity of inoculum dynamics seen in natural biofilm formation. This added sophistication enables them to better mimic in vivo conditions [46,47]. Such methodological distinctions probably account for the notable differences in PTE concentrations observed between our study and previous reports. Importantly, our study demonstrated that PTE effectively reduced the viability of streptococci, *mutans* streptococci, and *C. dubliniensis* within biofilm microcosms at concentrations of 160, 320, and 640 µg/mL, respectively. To our knowledge, this is the first report describing the antimicrobial activity of PTE against microcosm biofilms and specifically targeting streptococci, *mutans* streptococci, and *C. dubliniensis*. PTE fully inhibited both *C. albicans* and *C. dubliniensis* planktonic cells at 32 µg/mL. However, as expected, higher concentrations of PTE were required to significantly reduce the viability of biofilms formed by both *Candida* strains. This finding highlights the challenges associated with biofilm treatment, as these structures are more resistant to antifungal therapies [9,45]. Even in this context, treatment with PTE at 640 µg/mL decreased the viability of mature biofilms formed by *C. albicans* and *C. dubliniensis* by approximately 50%.

Given the in vitro antifungal effects of PTE against *Candida* spp. planktonic cells and their antimicrobial activity in microcosm biofilms associated with denture stomatitis, we extended our investigation to an in vivo experimental candidiasis model using *G. mellonella* larvae. The *G. mellonella* model has been widely recognized as a suitable system for evaluating drug toxicity and the antimicrobial efficacy of novel compounds, yielding consistent results with mammalian models [48,49]. Moreover, its low cost and ease of handling allow for high-throughput testing, making it an efficient alternative to conventional animal models [50]. In our study, PTE exhibited no toxicity toward *G. mellonella* larvae at concentrations up to 640 μg/mL. Supporting our findings, Yang et al. [51] demonstrated the biocompatibility of PTE with mammalian cells, showing no cytotoxic effects on keratinocytes (HaCaT) and human neutrophils at concentrations up to 256 μg/mL. However, mild toxicity was observed at higher concentrations of PTE. In addition, Li et al. [30] reported that PTE was non-toxic to mice at 64 μg/mL, reinforcing its safety profile across biological systems.

Since PTE was not toxic for *G. mellonella* larvae, we assessed its effectiveness using both prophylactic and therapeutic approaches. Larvae pre-treated with PTE exhibited increased survival and reduced early mortality. In contrast, therapeutic administration of PTE did not influence larval survival outcomes. Notably, Rossoni et al. [52] previously demonstrated that the pre-treatment of *G. mellonella* with extracts from *Lacticaseibacillus paracasei* 28.4 (formerly *Lactobacillus paracasei*) prolonged larval survival in larvae experimentally infected with *Candidozyma auris* (formerly *Candida auris*), through mechanisms involving direct antifungal activity and enhanced immune cell recruitment.

Based on these observations, we hypothesized that PTE might exert both antifungal and immunomodulatory effects in vivo. Prophylactic treatment with PTE significantly reduced the fungal burden of *C. albicans* in the hemolymph—an effect not observed in larvae infected with *C. dubliniensis*. This finding underscores the importance of investigating candidiasis caused by non-*albicans Candida* species, given their rising prevalence in clinical settings [53]. Regarding immunomodulation, pre-treatment with PTE did not influence the recruitment of hemocytes—key immune cells in the hemolymph involved in phagocytosis, nodulation, pathogen encapsulation, coagulation, and melanization [54]. These findings suggest that the enhanced survival observed during *C. albicans* infection may be primarily attributed to the antifungal properties of PTE rather than its influence on innate immune responses. However, further studies are warranted to investigate differences in virulence factors between the strains used, including filamentation capacity, adhesion, invasion and enzymatic activity, which may help explain the in vivo outcomes.

In summary, PTE demonstrated antifungal activity against planktonic cells of *C. albicans* and *C. dubliniensis*, as well as broad-spectrum antimicrobial effects on microcosm biofilms associated with denture stomatitis, effectively reducing the viability of staphylococci, streptococci, and yeasts. In vivo assays confirmed the biocompatibility of PTE in *G. mellonella* larvae. Furthermore, prophylactic treatment with PTE enhanced larval survival, likely due to a reduction in fungal burden within the hemolymph during *C. albicans* infection. However, further studies on PTE are needed to elucidate its broad-spectrum mechanism against non-albicans *Candida* species using a large number of clinical strains, particularly *C. dubliniensis*, which is a clinically relevant species in oral candidiasis.

## Figures and Tables

**Table 1:** Minimum inhibitory concentration (MIC; µg/mL), minimum fungicidal concentration (MFC; µg/mL), and MFC/MIC ratio values of pterostilbene (PTE) and fluconazole (FCZ) on *Candida* spp.

Strains	MIC (µg/mL)		MFC	MFC/MIC
	PTE	FCZ	PTE	PTE
*Candida dubliniensis* P1	32	0.25	32	1
*Candida albicans* P2	32	0.25	32	1
*Pichia kudriavzevii* ATCC 6258	32	16	ND	ND

ND: not determined.

## Data Availability

The original contributions presented in this study are included in the article. Further inquiries can be directed to the corresponding author.
